# Genome-wide analysis and visualization of copy number with CNVpytor in igv.js

**DOI:** 10.1093/bioinformatics/btae453

**Published:** 2024-07-17

**Authors:** Arijit Panda, Milovan Suvakov, Helga Thorvaldsdottir, Jill P Mesirov, James T Robinson, Alexej Abyzov

**Affiliations:** Department of Quantitative Health Sciences, Center for Individualized Medicine, Mayo Clinic, Rochester, MN 55905, United States; Department of Quantitative Health Sciences, Center for Individualized Medicine, Mayo Clinic, Rochester, MN 55905, United States; Broad Institute of MIT and Harvard, Cambridge, MA 02142, United States; Department of Medicine, University of California San Diego, La Jolla, CA 92093, United States; Moores Cancer Center, University of California San Diego, La Jolla, CA 92037, United States; Department of Medicine, University of California San Diego, La Jolla, CA 92093, United States; Department of Quantitative Health Sciences, Center for Individualized Medicine, Mayo Clinic, Rochester, MN 55905, United States

## Abstract

**Summary:**

Copy number variation (CNV) and alteration (CNA) analysis is a crucial component in many genomic studies and its applications span from basic research to clinic diagnostics and personalized medicine. CNVpytor is a tool featuring a read depth-based caller and combined read depth and B-allele frequency (BAF) based 2D caller to find CNVs and CNAs. The tool stores processed intermediate data and CNV/CNA calls in a compact HDF5 file—pytor file. Here, we describe a new track in igv.js that utilizes pytor and whole genome variant files as input for on-the-fly read depth and BAF visualization, CNV/CNA calling and analysis. Embedding into HTML pages and Jupiter Notebooks enables convenient remote data access and visualization simplifying interpretation and analysis of omics data.

**Availability and implementation:**

The CNVpytor track is integrated with igv.js and available at https://github.com/igvteam/igv.js. The documentation is available at https://github.com/igvteam/igv.js/wiki/cnvpytor. Usage can be tested in the IGV-Web app at https://igv.org/app and also on https://github.com/abyzovlab/CNVpytor.

## 1 Introduction

Copy number variation (CNV) and alteration (CNA) analysis plays a crucial role in identifying genomic alterations in diseases, understanding tumor heterogeneity, predicting treatment response, and investigating evolutionary biology and genetic diversity. Methods based on next generation sequencing data use read depth (RD) and B-allele frequency (BAF) information extracted from alignment (i.e. BAM) and variant call (i.e. VCF) files to estimate copy number (CN) level (Carter *et al.* 2012, Zaccaria and Raphael 2021). Raw data and CNV/CNA calls are often (particularly in clinical setting) visually inspected to assess possible artifacts due to incomplete reference assembly, repeat rich regions, and sequencing quality (Marshall *et al.* 2020). Reads are also checked at the call breakpoints to confirm the calls and spot complex events (Jin *et al.* 2019). The resolution of CNVs of different lengths depends on bin sizes used in the analysis, and flexible inspection of data using smaller and larger bin size is necessary (Xi *et al.* 2016).

Rapid growth of computing technologies enabled utilization of cloud computing resources to store and analyze the multi-modal omics data with instant visualization at local backend systems. Integration of multiple omics datasets helps researchers to infer complex regulation underlying biological processes and diseases. Visualization of such datasets is important for scientific interpretation. Genome viewers, like Integrative Genomics Viewer (IGV) (Robinson *et al.* 2011) and JBrowse (Diesh *et al.* 2023), have become a daily companion for understanding data for many organisms and have multiple tracks dedicated to specific data types. They come with various features including easy navigation, integration, and remote access to the input data. While tracks for displaying information for point mutations and variations are common, tracks that support instant visualization of RD signal, BAF signal, and CNV/CNA calls are still rare. For example, existing in igv.js Segmented Copy Number Track only allows visualizing precomputed copy number segments for deletions and duplications.

CNVpytor (Suvakov *et al.* 2021) is a tool designed to detect and analyze copy number (CN) from read depth and allele imbalance. It shares core functionalities with the predecessor tool, CNVnator, and incorporates many novel features including 2D calling methods to improve CNV/CNA detection accuracy (Suvakov *et al.* 2021, Bae *et al.* 2022). CNVpytor stores processed data from BAM and VCF files in HDF5 format, allowing for easy data access and further downstream analysis. Here, we have developed a new CNVpytor track in igv.js, the embeddable JavaScript implementation of the IGV (Robinson *et al.* 2023) for visualizing RD and BAF signals from fetching CNVpytor pre-processed CN-relevant data as well as for on-the-fly CN analysis, CN segmentation, CNV/CNA calling, and visualization directly from whole genome VCF file.

## 2 Features

**Figure 1.**
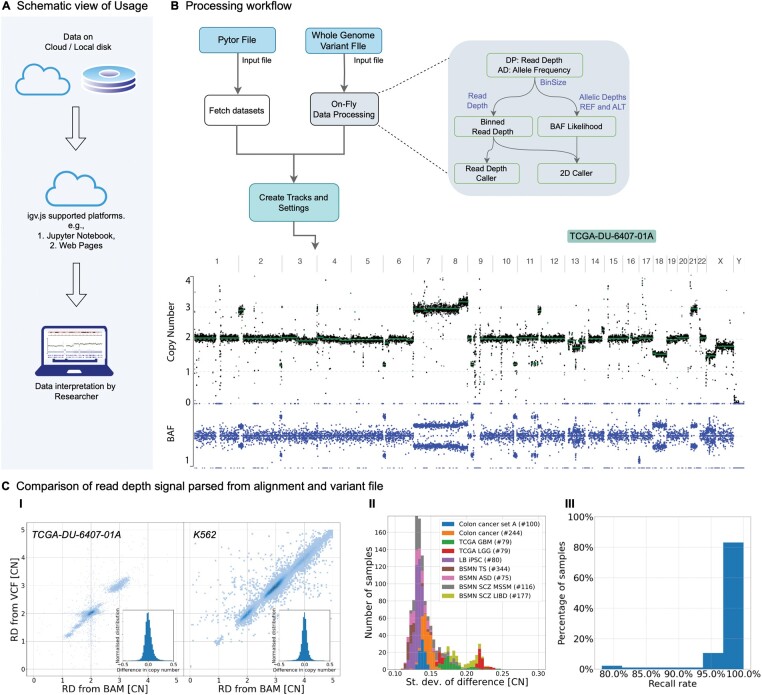
CNVpytor track usage. (A) Schematic view of data storage and analysis/visualization on a cloud accessible from the local researcher’s system. (B) Data processing workflow and CNVpytor track in igv.js with the view of copy number and BAF profiles for TCGA low grade glioma sample TCGA-DU-6407-01A. Multiple copy number alterations are visible and supported by splits in BAF likelihoods. (C) Comparison of read depth signal parsed from corresponding alignment (i.e. BAM) and variant (i.e. VCF) files. (I) For sample TCGA-DU-6407-01A and K562, 10 kb binned read depth was calculated and plotted on X and Y axis. Both samples show high concordance of RD calculated by each approach. (II) Comparison of read depth difference from VCF and BAM files for various cohorts shows that the difference typically does not exceed 0.2 CN. (III) Per sample distribution of the recall rate, defined as the percentage of events identified using 2D caller where read depth information is taken from the VCF file as compared to BAM file.

The igv.js can be embedded into HTML page or Jupyter Notebook. In igv.js, the CNVpytor track can be initiated by directly loading a pytor or VCF file, providing a URL to such a file through the menus, or specifying a track type with a link to a file in the relevant configuration. The track has two optional panels: one to display RD signal and one to display BAF signal (Fig. 1). The panel for RD can show up to 3 overlapping signals at a time: binned raw RD, binned GC corrected RD, and segments of CNV/CNA calls. If pre-calculated in the pytor file, CN segmentation and calls displayed can be from either RD or 2D callers. The latter uses both RD and BAF information and is set by default. BAF panel shows the maximum of BAF likelihood in each bin. Options are available to switch visualization between the callers, bin sizes, and signals. Viewing panels can render the whole genome view, a specific region, or multiple genomic loci simultaneously and to highlight a region (Supplementary Fig. S1).

CNVpytor stores the processed data in a HDF5 pytor file with a specific storage structure. CNVpytor track fetches and visualizes the chromosome-wide stored signals in the file. Alternatively, CN information can be parsed from VCF files and segmented on-the-fly (Fig. 1B). Whole genome VCF files are small as compared to whole genome alignment BAM files. The files can be further reduced by keeping the minimal necessary information for variants, i.e. read depth (DP) and allele depth (AD) fields. The track calculates RD and BAF signals for a bin size using values in those fields. The track implements the same callers as in CNVpytor: a mean-shift RD-based caller that was originally developed in CNVnator (Abyzov *et al.* 2011) and RD- and BAF-based 2D caller (Suvakov *et al.* 2021, Bae *et al.* 2022). By default, the 2D caller is used, but that behavior can be changed in the track configuration.

## 3 Utility of RD extraction from variant file

We compared read depth and CNV/CNA calls made using information extracted from matching VCF and BAM files for multiple samples and observed high concordance (Fig. 1C, Supplementary Fig. S2). There were a total of 1294 samples from multiple cohorts with coverage ranging from 3× to 440× (Supplementary Table S1). The distribution of differences in RD extracted from the matching files was centered around zero even for high CN, was symmetrical, and its standard deviation was always <0.25 of CN. With increasing sequence coverage, the standard deviation of the difference typically became smaller, however, lower sequencing quality also led to higher deviation (Supplementary Fig. S2A). We also directly compared calls made using signals obtained from BAM and VCF files. A few samples with lower quality of the data (i.e. with genome-wide BAF split) were excluded from the comparison. In over 80% of the samples, all CNV/CNA calls matched regardless of which file (BAM or VCF) was used to extract read depth information. For remaining samples, at least 80% of the calls matched. There was 90% of concordance in CNV type (Supplementary Fig. S2B).

## 4 Use case

A whole genome alignment (BAM) file was downloaded from TCGA for a low-grade glioma sample TCGA-DU-6407-01A. Germline variant calling was done by GATK HaplotypeCaller (DePristo *et al.* 2011). Given that somatic mutations typically constitute tiny fraction of the germline variants, using germline calls without additional filtering of somatic mutations is generally acceptable. Alignment and variant files were processed using CNVpytor using three bin sizes (10k, 100k, 1000k) to create a pytor file, which was then loaded into the igv.js viewer (Fig. 1). This cancer sample had many CNAs, including deletions, duplications and copy number neutral loss of homozygosity. A subclone encompassing duplications and deletions at ∼6% cell frequency was apparent (Fig. 1, Supplementary Fig. S1). Similar results can be obtained by on-the-fly loading and processing of the whole genome VCF file.

## 5 Conclusion or discussion

There are various approaches for calling CNVs and CNAs, each with its own structure for documenting processed data and calls. Currently, there is no widely accepted standard for storing such data, making accessing and visualizing the raw data used in CNV/CNA calling processes challenging. The CNVpytor track in igv.js provides enhanced functionality for the analysis and inspection of CNVs across the genome. By offering the ability to parse VCF files for both RD and BAF signals, CNVpytor and its corresponding track in igv.js provide a certain degree of standardization for inspecting raw data. In the future, developing a standard format for inspecting raw signals and converting outputs from various callers into such a format would be ideal. This track is a step toward that goal, making the scientific interpretation of research and clinical genomic data easier. For instance, primary data for cloud-based automated workflows for CNV/CNA calling can be instantly visualized and inspected on demand in a browser. Future developments will include parsing CNV/CNA-relevant data from other analysis tools and adding functionality to retrieve data for local genomic regions from CNVpytor files to enhance visualization speed.

## Supplementary Material

btae453_Supplementary_Data

## Data Availability

No new sequencing data were generated. Data for HepG2 and K562 cell lines were previously published (PMID: 30864654 and 30737237). Sequencing data for sample TCGA-DU-6407-01A is available through TCGA. Data used in figures 1C and S2 were taken from multiple cohorts, and details are provided in the supplementary table 1.
