# Supplementary material for: Genome-wide analysis and visualization of copy number with CNVpytor in igv.js
Source: Bioinformatics. 2024 Jul 17;40(8):btae453. doi: 10.1093/bioinformatics/btae453 (PMC11303504; doi:10.1093/bioinformatics/btae453)
Supplement: btae453_Supplementary_Data [file btae453_supplementary_data.zip › OP-CBIO240459_PECorr_AttachmentsFolder_SupplementaryFigures[AU].docx]

**Supplementary Figures**


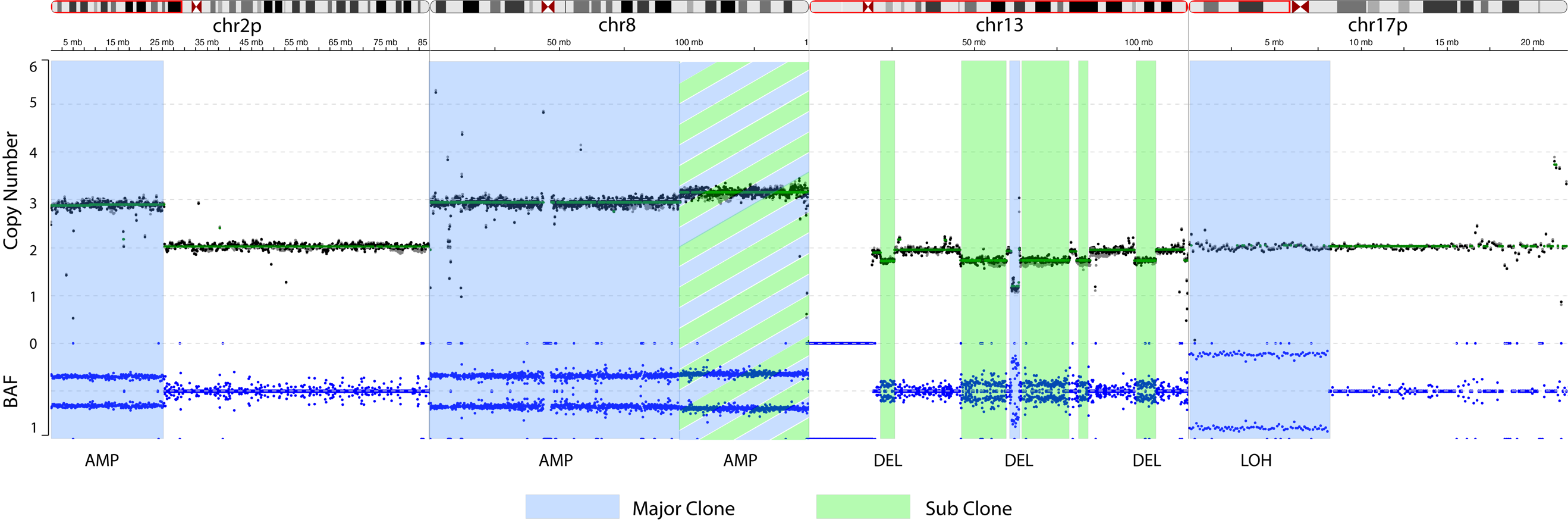


**Figure S1. LOH, clonal, and subclonal copy number alterations (CNA) in cancer sample TCGA-DU-6407-01A.** Four chromosomes are shown using multi-locus view in CNVpytor track in igv.js. CNAs belonging to different clones are highlighted with differ colors. The end of chromosome 8 has CNAs in both the major clone and a subclone. AMP – amplification, DEL – deletion, LOH – loss of heterozygocity.

**Figure S2. Comparison of CNA calls made using RD from corresponding BAM and VCF files. A)** Correspondence of the standard deviation of copy number differences with coverage. The standard deviation gets smaller with higher sequencing coverage. **B)** CNA event type-based comparison of calls. CNAs at low frequency the type can be discordant in their assigned type, but typically the called regions are concordant (see also **Fig. 1C**).
